# Towards Optical Biopsy in Glioma Surgery

**DOI:** 10.3390/ijms26104554

**Published:** 2025-05-09

**Authors:** Konstantin S. Yashin, Vladislav I. Shcheslavskiy, Igor A. Medyanik, Leonid Ya. Kravets, Marina V. Shirmanova

**Affiliations:** 1 Department of Neurosurgery, Privolzhsky Research Medical University, 10/1, Minin and Pozharsky Sq., 603950 Nizhny Novgorod, Russia; 2 Nizhny Novgorod Regional Oncological Hospital, 11/1 Delovaya St., 603093 Nizhny Novgorod, Russia; 3 Research Institute of Experimental Oncology and Biomedical Technologies, Privolzhsky Research Medical University, 10/1, Minin and Pozharsky Sq., 603950 Nizhny Novgorod, Russia; vis@becker-hickl.de (V.I.S.);

**Keywords:** intraoperative imaging, optical coherence tomography, OCT, FLIM, confocal endomicroscopy, Raman, brain cancer, glioblastoma

## Abstract

Currently, the focus of intraoperative imaging in brain tumor surgery is beginning to shift to optical methods such as optical coherence tomography (OCT), Raman spectroscopy, confocal laser endomicroscopy (CLE), and fluorescence lifetime imaging (FLIM). Optical imaging technologies provide in vivo and real-time high-resolution images of tissues. “Optical biopsy” can be considered as an alternative to traditional approaches for intraoperative histopathologic consultation. Intraoperative optical imaging can help to achieve precise intraoperative identification of tumor infiltrations within the surrounding brain parenchyma. Therefore, it can be considered as a complement to existing approaches based on wide-field imaging modalities such as MRI, US, or 5-ALA fluorescence. A promising future direction for intraoperative guidance during brain tumor surgery or stereotactic biopsy lies in the integration of optical imaging with machine learning techniques, enabling automated differentiation between tumor tissue and healthy brain parenchyma. We present this review to increase knowledge and form critical opinions in the field of using optical imaging in brain tumor surgery.

## 1. Introduction

Astrocytomas (gliomas) are the most common primary brain tumors with poor prognosis [1]. The most common histologic types in adult patients include glioblastoma (GBM) (grade 4), astrocytic tumors (grade 2–3), and oligodendroglioma (grade 2–3). With recent discoveries in genetics, the astrocytomas are divided into three main molecular groups based on the presence of IDH mutation [1]. IDH mutations are highly prevalent in gliomas and confer significant improved survival when compared to the IDH wild-type glioma [2,3]. Another key molecular alteration in astrocytoma is the codeletion 1p19q. It was demonstrated that patients with tumors lacking 1p and 19q were surviving longer and had benefited from the addition of chemoradiotherapy [4]. Therefore, from a clinical point of view, the three most common diffuse gliomas of adults should be distinguished: Glioblastoma IDH wild-type (which represents approximately 65–70% of adult glioma); Astrocytoma IDH mutant, 1p/19q non-codeleted (representing approximately 20–25%); and Oligodendroglioma IDH mutant, 1p/19q codeleted (5–7%) [1,5].

Despite a large number of innovations in neurooncological treatment options, the survival time often remains poor: low-grade gliomas have 5-year survival rates as high as 80%, while high-grade gliomas have 5-year survival rates under 5% [6]. There are several factors that determine unsatisfactory treatment results. To name a few, these are the infiltrative growth into surrounding brain tissue, localization near so called eloquent brain areas and special biological properties.

Although it is known that glioma cells can be found throughout the brain, up to 80% of patients develop initial tumor recurrence in close proximity to the resection site [7,8]. The main reasons seem to be the specific morpho-functional changes in peritumoral brain area and higher tumor cell density around the resection cavity [9,10]. Therefore, the role of surgery is crucial in the treatment of astrocytomas, and several studies have demonstrated the association between overall survival and the extent of resection [10,11,12,13,14]. However, the resection of astrocytomas is not possible within the classical oncological boundaries due to the presence of eloquent brain areas responsible for core brain functions, such as movement and speech. The modern paradigm of brain tumor surgery is based on an “onco-functional balance” principle—maximal safe resection preserving cortico-subcortical functions [15,16].

Intraoperative technologies are being developed in accordance with the two components of the concept of “onco-functional balance” [17]. On the one hand, functional MRI and DTI-fiber tractography can be used for preoperative building of highly detailed neuroanatomical and neurofunctional maps of the human brain [18,19,20]. Also, intraoperative neuromonitoring during awake or asleep surgery is the “gold standard” for preserving eloquent brain areas [18,21,22]. On the other hand, the technological enhancements in intraoperative imaging techniques have led to improved identification and visual discrimination of the tumor–brain margin: intraoperative magnetic resonance imaging (iMRI), intraoperative ultrasound (iUS), fluorescence imaging using 5-aminolevulinic acid (5-ALA), and sodium fluorescein have been shown to increase the extent of resection [14,23,24,25,26,27]. Although these methods have demonstrated high significance to identify residual tumor tissue and increased the extent of surgical resection, they are insufficient in the era of molecular profiling. The focus of intraoperative imaging begins to shift towards high-resolution optical methods, such as confocal and two-photon microscopies, optical coherence tomography (OCT), Raman spectroscopy, and fluorescence lifetime imaging (FLIM) [28,29,30]. By providing the neurosurgeon with information about tissue microstructure and biomolecular properties, optical technologies go beyond ‘the gold standard” of histopathological examination. Here, we present an overview of the major clinical applications of optical technologies that are relevant for brain tumor surgery. These technologies provided intraoperative visualization and gave an access to the unique information about the biological properties of the tumors, helping neurosurgeons to make the right decisions.

## 2. Basic Principles of Methods Used for Optical Biopsy

Recent advances in biomedical optical imaging make the term “optical biopsy” increasingly valid for clinical use. ‘Optical biopsy’ is an optical technique enabling the medical community to diagnose disease without removing a tissue sample [31]. Later, it was extended to the samples that have been removed from the patients [32]. The term was first used in 1984 by Alfano et al., who measured autofluorescence spectra of malignant and non-malignant human breast and lung tissues for the first time [31,33]. Further study of the mechanisms of tissue–light interaction led to the emergence of a large number of optical bioimaging techniques, each of which could detect the biological properties of tissues and differentiate tumorous and non-tumorous tissue with a certain diagnostic accuracy. In the surgery of gliomas, the most promising methods that have shown high sensitivity and specificity were confocal microscopy or confocal laser endomicroscopy (CLE) [34,35,36], Raman spectroscopy and Raman scattering histology [37,38], fluorescence lifetime spectroscopy and FLIM [39,40,41], and OCT [42].

Confocal microscopy is an optical technique that provides higher spatial resolution than conventional wide-field microscopy by blocking out-of-focus light with a confocal pinhole. During a scan, only one spot is imaged within a sample, and the whole image is constructed via point bypoint movement of a laser beam or a sample. Not only planar images but also in-depth optical sections can be collected with confocal microscopy, which gives an advantage of investigation of thick tissue samples and their 3D reconstruction. In neurosurgery, confocal microscopy is mainly used in the form of confocal laser endomicroscopy systems that enable intraoperative access to the brain via the miniaturized handheld probe. In confocal imaging, optical contrast of glioma tissue is typically achieved by the use of fluorescent dyes or precursors. The most frequently used dyes are sodium fluorescein [43,44] and 5-ALA-induced protoporphyrin IX [45,46], although other dyes, including methylene blue, acridine orange, indocyanine green, and acriflavine, have also been suggested as contrast enhancers. Note that excitation and emission spectra of most of these dyes are in the red spectral range, which allows for avoiding strong contribution from endogenous fluorescence.

Two-photon excitation microscopy (TPM) is an advanced version of confocal microscopy that exploits the effect of two-photon light absorption by the fluorophore to provide deeper tissue imaging and reduce photodamage of cells. Unlike standard confocal microscopy, TPM tends to explore endogenous contrast between gliomas and normal tissues using cellular autofluorescence, which is easily excited in the two-photon mode [47,48]. In general, TPM systems are technically complex and have historically relied on tunable femtosecond pulsed lasers that are expensive, limiting their adoption in the clinics. As a result, current TPM studies of patients’ gliomas are limited to the examination of fresh ex vivo tissue samples or slides using stationary setups. Although some progress has been made in the development of two-photon fluorescence endomicroscopes [49], they are still far from clinical use.

FLIM is based on the registration of fluorescence decay time (lifetime), the average time the fluorophore remains in the excited state. The fluorescence lifetime is specific for each fluorophore and largely depends on its molecular environment but, to a certain extent, does not depend on its concentration, which makes FLIM a quantitative technique in complex systems like tissues. The interest in FLIM as an optical biopsy method is driven mainly by its capability to record endogenous fluorescence, first of all, emitted by the NAD(P)H and flavins [41,50]. Gliomas reprogram many metabolic pathways of cells to ensure active proliferation, rapid growth, and adaptation to unfavorable conditions, including hypoxia and nutritional deficiency. The changes concern, first of all, energy metabolism but also affect biosynthetic processes and the regulation of redox balance. In glioma cells, the intensities of glycolysis, both anaerobic and aerobic (Warburg effect), glutaminolysis, and β-oxidation of fatty acids, are increased. In addition, IDHm gliomas have different metabolism patterns than IDHwt gliomas, which correlate with different survival outcomes [51,52,53,54]. Metabolic reprogramming of tumor cells inevitably affects the ratio of various forms of NAD(P)H and flavin cofactors that have different fluorescence lifetimes depending on binding to proteins. Therefore, images obtained using “label-free” FLIM contain unique molecular information, a biochemical map of the sample. Currently, FLIM systems based on two-photon fluorescence microscopy, macroscale confocal imaging, and fiber optic technologies are available, and all of these modalities have been tested for diagnosis of gliomas [55,56,57,58,59,60,61].

Raman spectroscopy is based on the process of inelastic light scattering, where photons lose part of their energy upon interaction with a molecule [62]. Measuring the energy lost during this interaction yields a spectrum that serves as a unique molecular fingerprint. Distribution of the lines in the Raman spectrum informs about the sorts of bonds in the molecule, allowing for identification of individual substances (e.g., aminoacids, proteins, lipids, nucleic acids, polysaccharides, etc.) Thus, Raman scattering enables highly accurate analysis of the chemical composition of biological samples by probing the vibrational energy levels of their constituent molecules. Compared to autofluorescence spectra, which result from the superposition of spectra of various molecules, Raman spectra are more specific, as they correspond to transitions between well-defined molecular vibrational levels [63]. Spontaneous Raman microscopy delivers submicron spatial resolution, but with a slow imaging speed due to a weak Raman signal [64,65]. By generating a much enhanced signal level, coherent anti-Stokes Raman scattering (CARS) or Stimulated Raman scattering (SRS) microscopy allows for video-rate vibrational imaging of biological samples [63].

OCT operates within the “biological transparency window” (wavelength range of 800–1500 nm), where light absorption is minimized, and penetration depth is maximized. These wavelengths fall outside the absorption bands of most tissue chromophores. As a result, light scattering at tissue interfaces, rather than absorption, is the primary contributor to OCT signal formation. The degree of scattering depends on the refractive index differences between cellular structures and the surrounding extracellular matrix. Since different cell types and tissue components exhibit distinct scattering properties, OCT can effectively differentiate between them, enabling high-resolution imaging of biological tissues. The white and gray matter of the brain exhibit distinct optical properties. White matter demonstrates significantly higher backscattering and absorption coefficients compared to gray matter [66,67,68]. This difference arises from their structural composition. Gray matter consists primarily of weakly scattering neuronal cell bodies, and white matter is predominantly composed of myelinated axons (70–95% of all fibers) [68], which strongly backscatter light due to their myelin sheaths [66,67]. The morphology of the tumorous tissue is characterized by the random nature of cell structures, variance in cellular nuclei sizes, and changes in the refractive index of the nucleus—cytoplasm, vascular proliferation, and areas of necrosis—which alter the nature of backscattering from a tissue [69].

Of these techniques, stimulated Raman spectroscopy and confocal microscopy are the most advanced methods in terms of the implementation into clinical practice [36,43,44,70]. A major advantage of Raman spectroscopy, OCT, and FLIM over other histological imaging techniques and confocal microscopy is that image contrast is generated by the intrinsic biochemical and structural properties of the tissue and does not require tissue processing or labeling. Despite the need for the use of contrast agents in confocal microscopy and the associated drawbacks, it has also been approved for clinical use [71].

## 3. Applications of Optical Biopsy in Glioma Surgery

Considering that there is no need to remove a tissue sample, optical methods can be used in the surgery of brain gliomas somewhat more widely than intraoperative histological examination: (1) as conventional histopathological examination—scanning of fresh specimens for fast determination of tissue type; (2) direct intraoperative imaging providing real-time feedback to the surgeons, e.g., clarifying the boundaries of the infiltrative brain tumors within surrounding tissues; (3) for guiding biopsy during stereotactic procedures.

### 3.1. Optical Biopsy for Intraoperative Histopathological Diagnosis

Intraoperative histopathological diagnosis seemed to be the most powerful tool in the determination of the tumor margins and the delivery of diagnostic information for the operating neurosurgeon. This can influence the course of the procedure—abort the operation or pursue aggressive surgical resection [72,73,74]. Intraoperative histopathological examination can be performed by the traditional method of frozen sections or recently introduced cytology smear [72,73,75]. For glioma, the overall diagnostic accuracy of frozen sections ranges from 78.4% to 95%, while that of cytology smears ranges from 50% to 100%; however, this difference is not statistically significant [72]. Despite the relatively high diagnostic accuracy of intraoperative consultations by pathologists, this approach has some disadvantages. First, it is time-consuming (30–40 min). Second, it lacks molecular information. Finally, it requires trained staff and expensive equipment [72,73]. The situation is significantly complicated by a shortage of board-certified neuropathologists, limiting the efficiency of such a pathologic consultation in neurosurgery [76]. Molecular subgrouping of diffuse gliomas requires laboratory techniques, such as immunohistochemistry, cytogenetic testing and next-generation sequencing, and long turnaround times (days–weeks) [77]. Intraoperative consultations can be improved by implementation of neural networks and artificial intelligence. They increase the speed of diagnostics, reduce pathology workload, and even provide indirect detection of key molecular signatures, including IDH and 1p19q [78]. The necessity for specimen preparation remains a time-limiting factor, suggesting that technologies enabling immediate tissue analysis would significantly accelerate intraoperative decision-making.

Label-free optical imaging technologies are of wide interest not only for neurosurgeons but also for pathologists, since many of them have so-called macroscopic versions [34,37,38,55,57,79] and allow for visualizing distinctly different and complementary information regarding brain tissue morphology (different types of cells, including neurons and glial cells, vascular proliferations, and necrosis, etc.) (Table 1). They provide the result much faster by reducing the time for sample preparation and owing to the introduction of machine learning methods for automatic image processing [34,80].

**Figure 1:**
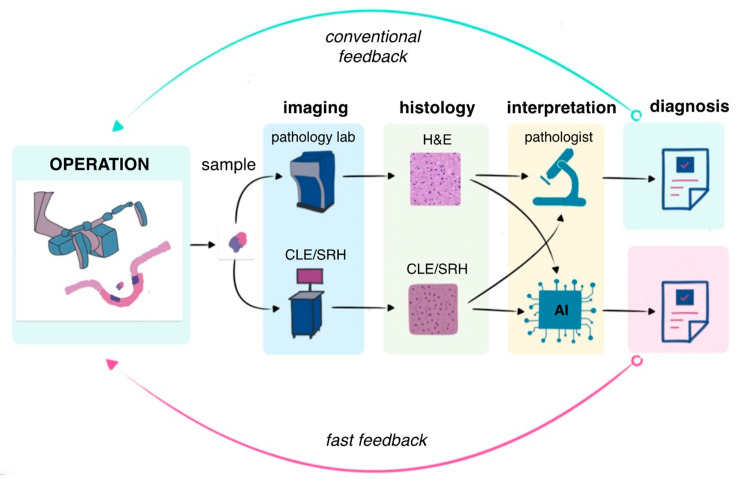
Optical biopsy for intraoperative diagnosis using CLE or SRH can provide fast feedback on tissue histopathological structure compared to conventional intraoperative diagnostics by frozen section or cytology smear. CLE—confocal laser endomicroscopy; SRH—stimulated Raman histology.

Most of the studies demonstrate that confocal microscopy and SRH correlate well with traditional histological findings, including the identification of many pathognomonic cytoarchitectural features of tumors and brain tissue [37,71,81,82,83]. Confocal microscopy captures hypercellularity, nuclear pleomorphism, and microvascular proliferation—hallmarks of GBM—with precision comparable to H&E staining [84]. SRH provides detailed visualization of both normal and pathological histological features [85]. In normal brain tissue, SRH clearly reveals characteristic elements such as neurons containing lipofuscin granules and the distinct appearance of lipid-rich axonal tracts. The technique also effectively identifies non-neoplastic pathological changes, including gliotic tissue remodeling and areas of macrophage infiltration. For astrocytomas, SRH demonstrates excellent capability in highlighting diagnostically important features such as perinuclear halos, tumor hypercellularity, nuclear atypia, and the presence of microvascular proliferation—all crucial for accurate tumor grading and classification [85]. Despite the fact that these methods make it possible to assess the morphological structure of the tissue without its preliminary processing, it is necessary to involve a pathologist to evaluate the resulting image. Thus, the techniques remain quite expensive for routine use in neurosurgery. The most promising solution to this problem is the use of machine learning for automatic classification of the tissue types for both methods [37,70,86]. Li et al. have demonstrated the application of the proposed deep learning framework to classify glioblastoma and meningioma brain tumors based on endomicroscopic data, achieving accuracy equal to 99.49% [86]. Another study by Hollon et al. has demonstrated non-inferior diagnostic accuracy compared to conventional pathology (94.6% vs. 93.9%) with significantly faster processing times (150 s vs. 20–30 min), suggesting potential to reduce reliance on intraoperative pathology consultation. However, this issue requires legal regulation. In general, the combination of optical methods and artificial intelligence seems to be the most promising for fast intraoperative diagnosis compared to conventional intraoperative histopathological evaluation (Figure 1).

OCT does not have sufficient resolution to detect tumor cell infiltration; however, it can provide the neurosurgeon with information about tissue properties that are not available by standard histopathological methods. For example, Assayag et at al. have acquired large size OCT images with spatial resolution comparable to histological analysis, sufficient to distinguish microstructures of the human brain parenchyma—myelin fibers, neurons, microcalcifications, tumor cells, microcysts, and vessels [79]. The advantages of the described system were relative compactness for use in the operating rooms and fast image acquisition. Using this system, it was possible to distinguish individual myelin fibers of 1 μm in diameter. This indicates the high sensitivity of OCT for white matter evaluation without any staining. However, astrocytoma infiltration could be detected on full-field OCT images only if the glial cell density was greater than 20%. Therefore, this system is not currently suitable for the evaluation of tissues with low tumor infiltration or tumor margins [79].

Several works demonstrate the possibility to distinguish glioma from normal tissue using time-resolved measurements of cellular autofluorescence with FLIM microscopy [39,40,41,87,88,89]. However, the potential of FLIM microscopy in detection of glioma cells at the infiltrative edge is poorly explored. This is, in part, due to the technical challenge to compare FLIM images acquired from fresh tissue samples and corresponding histological slices. In general, imaging of gliomas using FLIM microscopy is complicated by high intratumoral heterogeneity and the lack of clear boundaries with peritumoral brain tissue. However, FLIM performed at the macroscopic scale presents a viable substitute for intraoperative frozen sections, allowing for real-time analysis of biopsy tissue samples (ex vivo) [41,90]. The initial data showed difference in NAD(P)H fluorescence decay between tumorous tissue and white matter in astrocytoma patients using macroscopic FLIM [41].

Therefore, it can be concluded that optical technologies for intraoperative histopathological diagnosis are being introduced to reduce the need for intraoperative consultation. Among these methods, SRH has emerged as especially significant. Research demonstrates that these technologies achieve diagnostic accuracy comparable to conventional histological examination while offering substantially faster turnaround times.

### 3.2. Optical Imaging for Neurosurgical Guidance

Despite the implementation of a combination of treatment options, recurrence is a common thing, with over 80% of cases arising at the edge of the resection cavity. The high recurrence rate can be explained by infiltrative growth into surrounding brain tissue with the formation of the so-called peritumoral zone. As compared to a healthy brain tissue, this area is characterized by a set of molecular, biochemical, radiological, and cellular specific features [91,92,93]. Tumor cells in the peritumoral zone possess high invasive capacity in contrast to the cells of the tumor core [94,95] and play a key role in tumor recurrence [96].

**Figure 2:**
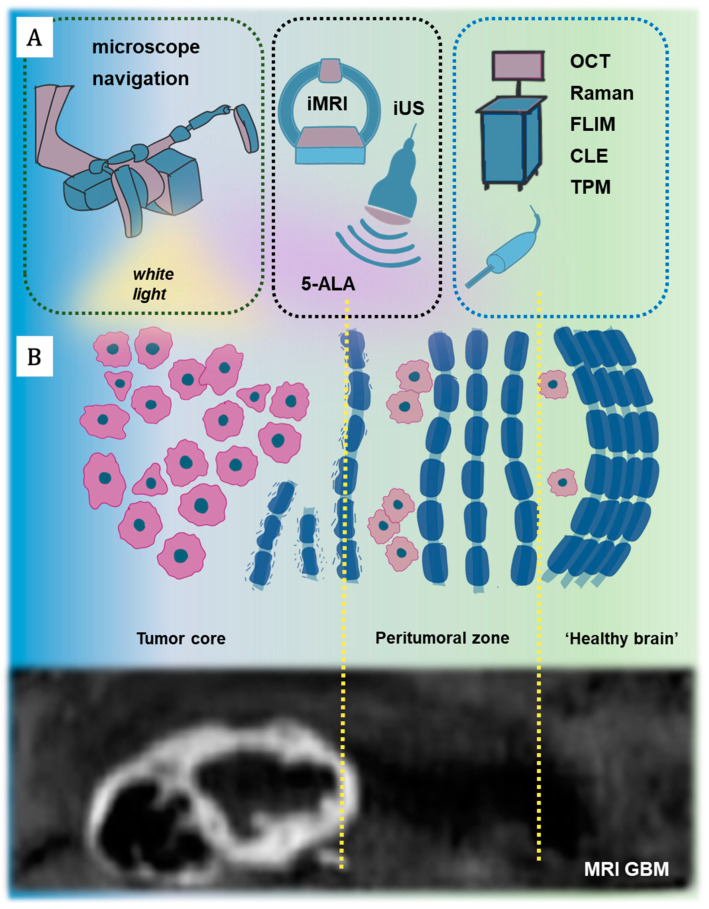
(**A**). Brain tumor diagnostics using a complex approach. iMRI—intraoperative MRI, iUS—intraoperative ultrasound, 5-ALA—5-Aminolevulinic Acid, Raman—Raman microscopy or spectroscopy, OCT—optical coherence tomography, FLIM—fluorescence lifetime imaging, CLE—confocal laser endomicroscopy, TPM—two-photon microscopy. (**B**). Schematic and MRI imaging of the different areas around a brain tumor: (1) “tumor core”—the main tumor mass which corresponds to the contrast-enhancing regions observed on MRI; (2) “peritumoral zone” is characterized by a set of molecular, biochemical, and cellular specific features and usually is depicted on MRI as an area of perifocal edema; (3) “healthy brain”, which consists of macroscopically healthy brain parenchyma that comprises solitary tumor cells.

Therefore, the main concept of using different intraoperative tools is to detect the peritumoral brain zone infiltrated by tumor cells and/or having the potential for further tumor growth directly during tumor removal by a neurosurgeon. All intraoperative tools can be divided into three main groups in accordance with the theoretical model, whereby the tumor microenvironment consists of at least three heterogeneous areas [97]. The use of neuronavigation and microsurgical tumor removal under the white light of a microscope allows for significant cytoreduction of the core tumor zone, which corresponds to the contrast-enhancing regions observed on MRI (Figure 2B). However, this combination is not sufficient for total tumor resection due to several reasons: (1) insufficient resolution—tumor resection in the white light of a microscope can only achieve a maximum resection in 23–50% of cases [98,99]; (2) absence of real-time intraoperative representations of the tumor and surrounding structures due to unpredictable brain shifts, distortions, and deformations [100]. The most relevant and widely used methods for intraoperative identification of both the tumor core and the peritumoral zone are intraoperative MRI (iMRI), intraoperative ultrasound (iUS), and 5-ALA or other fluorescence agents [24,101,102,103] (Figure 2A).

The major advantage of using these methods is that they provide real-time information about the structure of the tissue that goes far beyond the capabilities of wide-field microscopy. However, there are several limitations of these methods. Some studies have demonstrated low sensitivity of 5-ALA fluorescence for low-grade astrocytomas [104,105]. Moreover, the assessment of fluorescence intensity is observer-dependent and thus subjective, which can result in the preservation of a part of the tumor tissue possessing low fluorescence [24,106]. iMRT has a number of disadvantages, including high cost, inability to integrate with the microscope, and the need to use special surgical tools. The technique has a long learning curve and requires a high level of training for surgeons [102].

Optical imaging methods have a significantly higher spatial resolution and, as a result, demonstrate higher sensitivity and specificity compared to conventional tools for detection of residual tumor cells in peritumoral zone (Table 2). A real-time in vivo analysis tissue structure at the microscopic level could contribute to a better and quicker visualization of the tumor border and inspecting eloquent tissue for tumor invasion. However, most optical technologies have strong limitations for being a standalone technology: (1) small penetration depth; (2) limited field of view; (3) the large size of used devices, which are not adapted for operation room workflow. However, for specific optical technologies, dedicated devices have been developed to enable intraoperative diagnostic imaging without disrupting the surgical workflow. Fundamentally, two distinct implementation approaches exist: (1) utilization of handheld imaging probes [107,108,109,110,111] or (2) integration of the technology as a modular component within the surgical microscope [112,113].

In addition, in Raman spectroscopy the weak signal intensity and challenges in data acquisition and processing restrict real-time applicability of this method [28].

Confocal microscopy has already demonstrated promising results in clinical trials; however, this method requires the use of contrast agents [35,43,44,46,82].

Label-free intraoperative delineation of the tumor margin can potentially be performed using multispectral time-gated spectroscopy with a fiber probe for hand-scanning, point-measurement imaging directly in patients [39,87]. However, this method can predict the tumor cell density in the infiltrative edge in real-time with relatively low accuracy and does not provide microscopic resolution to identify single cells [39,87].

Therefore, taking into account all the advantages and disadvantages, optical technologies may be regarded as a supplementation of traditional imaging techniques [114]. For example, in a resection cavity margin where iMRI, ultrasound, or fluorescence suggests the presence of tumor, or if they offer conflicting results, high-resolution optical imaging can provide a quick and reliable assessment of tumor cells infiltration. Combined use of iUS and 5-ALA fluorescence with high-resolution imaging may ultimately impact extent of resection, but clinical data supporting this hypothesis have not yet emerged.

The most striking example of such a combination is the use of quantitative spectroscopic analysis of 5-ALA-induced PpIX accumulation [105,109,115,116]. Quantitative PpIX analysis represents a powerful technique for improved intraoperative detection of low-grade astrocytoma that is generally characterized by the absence of visible fluorescence in order to maximize the extent of resection [117,118,119]. Implementation of spectroscopic analysis has also demonstrated a significant correlation between fluorescence intensity and proliferation rate [106].

A comparative study of Raman spectroscopy and 5-ALA fluorescence predicts their high diagnostic accuracy when used together, while Raman is capable of detecting tumor-infiltrated brain with higher sensitivity (69% vs. 46%) but lower specificity (57% vs. 81%) comparing to fluorescence [120]. The CLE during 5-ALA-guided glioma surgery also improves diagnostic accuracy. Recent studies of 5-ALA wide-field imaging and CLE in nine patients with gliomas have demonstrated a sensitivity/specificity of CLE and 5-ALA for the interpretation of tumor margins of 79%/67% and 50%/67%, respectively [110]. Abramov et al. have demonstrated CLE has higher accuracy in detecting regions with infiltrating tumors than 5-ALA imaging [109]. The sensitivity and specificity of CLE for the interpretation of tumor margins were 73% and 41%, respectively, and those of 5-ALA were 38% and 82%, respectively [109]. In addition, a pilot study indicates that combined FLIM of NADH/PpIX represents a sensitive tool to visualize brain tumor tissues that are not detectable with conventional 5-ALA fluorescence [57]. One has to mention that there have been no attempts to combine TPM and OCT for brain tumor diagnostics [47].

Thus, the intraoperative application of optical imaging technologies significantly improves the diagnostic accuracy of differentiating neoplastic from non-neoplastic tissue at the microscopic level. A critical aspect of the application of optical technologies is their ability to support real-time surgical decision making during tumor resection. Clinical validation studies have demonstrated that CLE, OCT, Raman spectroscopy, and FLIM achieve diagnostic accuracies exceeding 90%, enabling more confident intraoperative determination of resection margins. Thus, optical technologies universally contribute to more precise tumor resection and volumetric control, irrespective of their particular analytical capabilities. However, a critical limitation of these modalities is their limited field of view, which is often restricted to localized regions of interest. Consequently, a predominant research direction focuses on the integration of these high-resolution optical techniques with wide-field imaging systems to enable comprehensive intraoperative margin assessment.

**Table 2:** Comparison of different conventional and optical technologies in surgical guidance of brain cancer.

Technology	iMRI	iUS	5-ALA	Raman	OCT	FLIM	CLE	TPM
Type of energy measured	radio waves	high-frequency sound waves	visible light	visible or near-infrared light	near-infra-red light	visible or near-infrared light	visible or near- infrared light	near-infrared light
Penetration level	organ-tissue	organ-tissue	tissue	cellular-molecular	tissue-cellular	tissue-cellular	tissue-cellular	tissue-cellular
Imaging application	whole brain	tissue surface/subsurface of tissue	tissue surface	tissue surface	tissue surface/subsurface of tissue	tissue surface	tissue surface	tissue surface/subsurface
Spatial resolution	20–100 um	50–500 um	0.03 mm	300 nm–1 μm	0.02 mm	300 nm–15 μm	300 nm–500 nm	300 nm–1000 nm
Time resolution	minutes to hours	seconds to minutes	seconds	seconds	seconds	ps-ms	seconds	seconds
Contrast enhanced	label free or small molecules nanoparticle	label free or microbubble	labeled	label free	label free	label free	labeled	label free or labeled
Cost	very high	moderate	moderate	high	low	high	moderate	high
Intraoperative tools	-	probes	probes or contactless	probes	probes or contactless	probes	contactless	contactless
Type of information	structural	structural	metabolic	“optical fingerprint”	structural	metabolic	structural	structural
Sensitivity (%)	41–96 for HGG	46–80 for LGG and HGG	91 for HGG	84–96 for LGG/HGG	85 for HGG 90 for LGG	58 for HGG	85–91 for HGG	100 for HGG
Specificity (%)	57–100 for HGG	28–100 for LGG/HGG	80–89 for HGG	89–100 for LGG/HGG	85 for HGG 90 for LGG	72 for HGG	81–94 for HGG	50 for HGG
GTR achieving	96–100%	73.4%	~76%	No data	No data	No data	No data	No data
References	[102,121,122,123]	[124,125,126,127]	[122,128,129,130]	[38,131,132,133]	[107,108,134,135,136]	[87]	[71,137]	[48,61]

iMRI—intraoperative MRI, iUS—intraoperative ultrasound, 5-ALA—5-Aminolevulinic Acid, Raman—Raman microscopy or spectroscopy, OCT—optical coherence tomography, FLIM—fluorescence lifetime imaging, CLE—confocal laser endomicroscopy, TPM—two-photon microscopy; GTR—gross total resection.

### 3.3. Optical Imaging for Stereotactic Biopsy Guidance

Currently, stereotactic biopsies are a routine neurosurgical procedure for astrocytomas and intracranial lymphomas that are not amenable to resection. Therefore, it is one of the most frequently performed brain surgeries [138]. The goal of the surgery is to obtain viable tissue representative of the lesion in order to provide a comprehensive histological analysis. However, there is a risk of acquiring non-diagnostic samples from outside of the viable tumor volume (such as necrotic/gliotic tissue or normal white matter), which has been reported in up to 24% of stereotactic biopsy series and suggested repeated neurosurgical interventions [139,140,141]. Therefore, the serial biopsies [142,143] and intraoperative consultation by an experienced neuropathologist [140,141,144] are commonly used to improve the diagnostic yield and accuracy of stereotactic biopsies. However, such an approach faces two problems: (1) intraoperative consultations are time-consuming, costly, and generally not permanently available [141,145,146]; (2) the acquisition of serial biopsies is associated with an increased risk of intracranial hemorrhages, which have been reported in 0.3–59.8% of cases [140,147,148] and contribute considerably to the reported morbidity and mortality [140,141,142,149,150]. Taking this into account, the development of the technologies is aimed at the reduction in the risks of complications [151].

**Figure 3:**
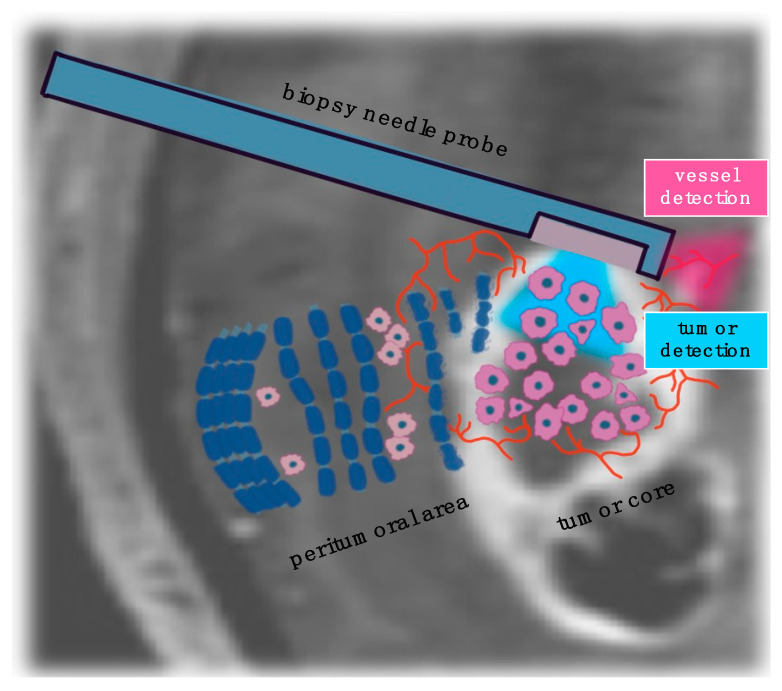
Optical imaging can be integrated in stereotactic biopsy needle probe for vessel detection and avoidance of vessel injury; tumor detection; and biopsy acquisition.

Several studies have demonstrated the clinical benefits of using 5-ALA fluorescence for tumor detection in tissue samples during stereotactic biopsy procedures [152,153,154,155,156]. The high spatial resolution and diagnostic accuracy for tumor detection indicate the feasibility of integrating optical imaging techniques directly into the biopsy probe needle via fiber optics (Figure 3), including Raman spectroscopy and 5-ALA/PpIX imaging [38,131,157,158]. Moreover, optical imaging technologies could improve the safety of procedure by detecting a small vessel along the trajectory (Table 3) and, therefore, decrease the risk of vessel damage and subsequent bleeding.

In general, optical imaging can be used in three different ways in stereotactic biopsy guidance [159]: (1) avoidance of vessel injury during stereotactic biopsies, (2) tumor detection and biopsy acquisition, and (3) rapid intraoperative assessment of stereotactic biopsy specimens.

**Table 3:** Comparison of different conventional and optical technologies for guidance during stereotactic brain cancer biopsy.

Technology	iMRI	iUS	5-ALA	Raman	OCT
Tumor detection	yes	yes	yes	yes	yes
Vessel detection	yes	yes	yes	no	yes
Sensitivity	-	-	63–69 for biopsy acquisition	80 for biopsy acquisition	91.2 for blood vessel
Specificity	-	-	100 for biopsy acquisition	90 for biopsy acquisition	97.7 for blood vessel
Diagnostic accuracy (%)	over 97 for biopsy acquisition	88.4–91.5 for biopsy acquisition	98 for biopsy acquisition	84 for biopsy acquisition	-
References	[102]	[160]	[161,162]	[38]	[161,163]

Göbel et al. conducted a clinical pilot trial on a minimally invasive probe that can be inserted into the tissue through a regular biopsy needle [164]. The same fiber optics is used for both illumination and image detection, enabling a clear tumorous tissue and vessel detection based on tissue autofluorescence, PpIX fluorescence, and indocyanine green (ICG) fluorescence. Another approach is to use the combination of 5-ALA fluorescence and a laser Doppler flowmetry system for the detection both the tumorous tissue and blood vessels [165].

OCT can also be integrated into a standard biopsy needle, giving the opportunity to guide the needle movement along trajectory and detect a vessel [163,166,167,168], as well as to perform direct analysis of the tissue structure in the biopsy area [169]. The combined OCT and fluorescence system has also been suggested [158].

Therefore, optical imaging is a new trend for improving patient safety and surgical workflow during stereotactic procedure for brain tumor biopsy. Future advances in machine learning and artificial intelligence can improve intraoperative real-time navigation via optical imaging.

## 4. Real-Time Molecular Characterization Using Optical Technologies: Transforming Intraoperative Surgical Strategies

In modern glioma surgery, determining the optimal extent of resection requires careful consideration of molecular subtype-specific prognostic factors alongside potential risks of neurological deficits when operating near eloquent brain regions. This balance between maximal tumor removal and functional preservation has become increasingly complex with our growing understanding of how molecular profiles influence both tumor behavior and response to surgical intervention.

The molecular profiling capabilities of these optical modalities are highly variable. Techniques such as OCT and CLE, while providing rapid intraoperative visualization of tissue and cellular architecture at high resolution, remain fundamentally constrained to morphological assessment. This inherent limitation may result in undetected high-grade tumor foci that exhibit molecular alterations without corresponding histological abnormalities. In contrast, Raman spectroscopy and FLIM offer superior potential for molecular characterization of gliomas, capable of providing data that extends beyond conventional H&E staining to reveal critical tumor biomarkers [40,170,171,172].

FLIM reveals metabolic alterations through NAD(P)H autofluorescence patterns, with IDH-mutant oligodendrogliomas demonstrating significantly shorter lifetimes (3.3 ± 0.1 ns) compared to IDH-mutant astrocytomas (4.1 ± 0.1 ns) [40]. Raman spectroscopy offers direct molecular specificity by detecting the pathognomonic oncometabolite 2-hydroxyglutarate (2-HG), which accumulates specifically in IDH1/2-mutant tumors and produces a unique spectral signature between 1130 and 1300 cm^−1^ [173].

Non-invasive prediction of molecular subtypes during surgical intervention is now challenging but may play a dramatic role in decision making. Surgical management appropriately focuses on the nuances of surgical resection: whether to target only the enhancing component of disease versus more extensive resection, including the non-enhancing component, and the use of molecular classification to tailor this decision making [174]. Extensive resection increased survival in specific patients with IDHm astrocytoma [175]. In IDHwt glioblastomas, some molecular subtypes may benefit from aggressive resections [176]. In oligodendrogliomas, extensive resection does not improve patients’ survival [177].

The development of rapid, non-invasive intraoperative techniques that provide precise molecular information would be a significant advancement in neurosurgical oncology, enabling real-time adaptation of surgical strategy to individual tumor characteristics.

## 5. Challenges and Future Prospects of Optical Technologies in Glioma Surgery

Current clinical experience demonstrates significant progress in developing optical diagnostic modalities for glioma resection. Multiple studies confirm the clinical potential of these techniques, offering several key advantages: (1) enhanced discrimination between neoplastic and normal parenchyma, (2) improved extent of resection, (3) increased accuracy and safety of stereotactic biopsies, (4) reduced operative duration, and (5) decreased costs associated with intraoperative frozen-section analysis. Most notably, certain technologies have obtained FDA clearance, such as, for example, exemplified CLE systems [137].

However, several barriers impede widespread clinical adoption, including (1) absence of standardized imaging protocols, (2) complex regulatory approval processes, and (3) substantial costs associated with advanced systems. Methodological challenges in study design are particularly noteworthy, as developing protocols for assessing complete resection remains problematic due to the subjective nature of image interpretation. Most investigations utilize diagnostic accuracy for tumor identification rather than volumetric resection assessment as primary endpoints. Comprehensive evaluation of resection completeness requires multimodal integration of MRI, wide-field fluorescence, and optical imaging data.

Practical implementation challenges include (1) difficulties maintaining rigorous study protocols during dynamic surgical decision-making and (2) resource limitations restricting multicenter participation. The most compelling evidence comes from SRH studies, where Hollon et al. demonstrated non-inferior diagnostic accuracy compared to conventional pathology (94.6% vs. 93.9%) with significantly faster processing times (150 s vs. 20–30 min), suggesting potential to reduce reliance on intraoperative pathology consultation [80].

Future development should focus on several key areas: (1) integration of hybrid optical systems combining structural, molecular, and/or metabolic data (e.g., OCT + Raman [178] or CLE + FLIM); (2) implementation of machine learning algorithms for real-time, objective image interpretation [179]; (3) standardization of clinical protocols based on developing consensus guidelines for image acquisition and analysis; and (4) optimizing systems for broader clinical accessibility. As these technologies develop, they may fundamentally transform glioma surgery by providing comprehensive intraoperative characterization of the tumor.

Current research evidence on the application of optical technologies in glioma surgery suggests that intraoperative diagnosis may soon be achieved through these modalities, significantly reducing conventional diagnostic processing times. In this emerging paradigm, various optical techniques will compete within the biomedical market, each striving to provide maximal surgical utility and clinical advantage.

## 6. Conclusions

Recent advances in biomedical optical imaging make it possible to diagnose disease without removing a tissue sample in routine practice. Optical imaging technologies have demonstrated advantages compared to conventional intraoperative tools in neurosurgery: high resolution, high speed, low cost, label free, non-invasiveness, and convenient performance. Providing a quick and reliable assessment of tumor cells infiltration or blood vessels, optical technologies may be regarded as a supplement for existing approaches based on wide-field imaging modalities, such as MRI, US, or fluorescence imaging.

The implementation of optical imaging for automated delineation of tumorous tissue from surrounding healthy brain parenchyma based on machine learning approaches seems to be the future of intraoperative guidance during brain tumor surgery or stereotactic biopsy. However, there are still limitations to be addressed (e.g., the lack of standardized protocols for some of its applications), and further large clinical trials are needed. In addition, the cost-effectiveness of these devices and the possibility of using them on a daily basis must be considered for widespread implementation of optical imaging. Some of the technologies, for example, 5-ALA, are already used in brain tumor surgery to increase the diagnostic yield, and an extension to the stereotactic biopsy procedures could be easily implemented. On the other hand, for SRH or CLE, which are already demonstrating amazing capabilities in non-invasive visualization of tissue structure, comprehensive studies must be carried out, including those justifying their cost-effect.

It should also be mentioned that some optical imaging techniques, e.g., Raman spectroscopy and FLIM, allow us to obtain not only structural but also molecular information, which can be potentially used to intraoperatively, on a label-free basis determine the molecular subtypes of the tumors. In the future, the integration of these methods into glioma surgery may significantly improve treatment outcomes.

## Figures and Tables

**Table 1:** Comparison of intraoperative consultation via frozen sections with optical technologies.

	Frozen Section	Stimulated Raman Histology	Full-Field OCT	Macroscopic FLIM	Confocal Microscopy	Two-Photon Microscopy
Label free	label free	label free	label free	label free	labeled	label free or labeled
FOV	10–20 mm	100 μm–1 mm	10 mm	20 mm	100 μm–1 mm	100 μm–1 mm
Lateral spatial resolution		300 nm	10 μm	15 μm	300 nm	500 nm
Time of diagnosis	~30–40 min	~2–10 min	no data	no data	no data	no data
Cost	High	High	Low	High	Moderate	High
Type of information	Morphology	Morphology, “optical fingerprint”	Morphology	Metabolism	Morphology and metabolism	Morphology and metabolism
Identify malignant cells	yes	yes	yes	no	yes	yes
Molecular information	no	yes	no	no data	no	no
Diagnostic accuracy for tumor identification	~78.4% to 95%	~90–100%	no data	no data	~80%	no data

## Abbreviations

The following abbreviations are used in this manuscript:

5-ALA	5-aminolevulinic acid
CLE	confocal laser endomicroscopy
EOR	extent of resection
GBM	glioblastoma
GTR	gross total resection
FLIM	fluorescence lifetime imaging
ICG	indocyanine green
IDH	isocitrate dehydrogenase
iMRI	intraoperative magnetic resonance imaging
iUS	intraoperative ultrasound
SRH	stimulated Raman histology
OCT	optical coherence tomography
TPM	two-photon microscopy
